# Greater auricular nerve neuropraxia with beach chair positioning during shoulder surgery

**DOI:** 10.4103/0973-6042.70824

**Published:** 2010

**Authors:** Albert K. H. Ng, Richard S. Page

**Affiliations:** Barwon Orthopaedic Research Unit, The Geelong Hospital, Geelong, Victoria 3220, Australia

**Keywords:** Beach-chair position, greater auricular nerve, neuropraxia, shoulder surgery

## Abstract

Neuropraxia of the greater auricular nerve is an uncommon complication of shoulder surgery, with the patient in the beach chair position. The greater auricular nerve, a superficial branch of the cervical plexus, is vulnerable to neuropraxia due to its superficial anatomical location. In this case series, we present three cases of neuropraxia associated with direct compression by a horseshoe headrest, used in routine positioning for uncomplicated shoulder surgery. We outline the risk of using devices of this nature and discourage the use of similar headrest devices due to the potential complications in headrest devices that exert pressure on the posterior auricular area to maintain head position during surgery.

## INTRODUCTION

Shoulder arthroscopy has been shown to be the procedure of choice for many diagnostic and therapeutic procedures over the past 20 years. As with all interventions, complications can occur, which need to be recognized, managed and minimized by the orthopedic surgeon. We report a total of three cases of resolved neuropraxia in the greater auricular nerve. In two of the cases, an arthroscopic approach was taken, while an open approach was used in one other case of surgery of the shoulder with the patient under general anesthesia in the beach-chair position.

## CASE REPORTS

### Case 1

A 43-year-old female underwent open reduction of the right acromioclavicular (AC) joint and stabilization of the right lateral clavicle under general anesthesia without shoulder regional anesthesia.

The standard beach-chair position, with the patient in an upright position at an angle of 45° to the floor, hips flexed at 60° and knees flexed at 30°, was used. The head was secured in a neutral position to a horseshoe-shaped headrest. Care was taken to protect the deep neurovascular structures throughout. The patient was in this position for the full 100 min of the operation, from draping to wound closure. Before draping the patient, care was taken to ensure that positioning of the head and neck was in the neutral position. Perioperatively, positioning was checked to ensure that no movement occurred. Postsurgical review on the ward the next morning did not reveal discomfort or neurological symptoms. On 2-weeks postsurgical review, however, she complained of tingling in her fingers and pain and stiffness in her neck and numbness in the distribution of the posterior auricular nerve. A neurological review was requested and obtained 8 weeks postsurgically, by which time her symptoms had completely resolved and the neurology and neurophysiology had returned to normal.

### Case 2

A 25-year-old right hand-dominant male underwent arthroscopic elective superior labrum anterior to posterior (SLAP) repair for a superior and posterior inferior labral tear.

General anesthesia and an interscalene block with 24 ml of 0.75% ropivucaine and 140 µg clonidine was used. The patient was set-up in the standard beach-chair position as outlined in Case 1. Standard prep and drape was used with 4.0 kg of longitudinal traction and the horseshoe head rest. Standard posterior and anterior working portals were used.

Findings were of extensive labral pathology and a large bony Hill Sach’s lesion was noted with a small bony anterior glenoid deficiency with deficient labral remnant.

Extra working portals were created and the posterior labrum and superior labrum were repaired arthroscopically with a range of bioabsorbable suture anchors.

The patient was thus in this position for the full 125 min of the set up and operation. Before draping the patient, the same care was taken to ensure that positioning of the head and neck was in the neutral position pre- and peri- operatively. In recovery, it was noted that the patient complained of pain in the posterior auricular distribution, which commenced about 30 min postoperatively.

Postsurgical review in the ward the next morning did not reveal discomfort. The patient was discharged the following day and seen at 2 weeks, with complaints of numbness and dysesthesia in the greater auricular nerve distribution on one side. This continued with reducing pain over 3 months, with the altered sensation resolving 6 months later without deficit.

### Case 3

An 18-year-old right hand-dominant male underwent an arthroscopic right shoulder SLAP repair, Bankart repair and inferior capsular shift.

Under general anesthesia, the patient was given IV antibiotics and set-up in the beach-chair position in the standard fashion. The patient was in this position for the full 120 min of the operation. Preparation and drape was used with an elbow brace and a total of 3.5 kg longitudinal traction. Standard posterior and anterior working portals with additional anterosuperior and posterior axillary pouch portals[1] were created.

Findings at arthroscopy were of a large Bankart defect. A shift was performed arthroscopically and stabilized with three bioabsorbable suture anchors.

On review the following day in the ward, there was good pain control. However, the patient was experiencing an area of numbness in the posterior auricular region. This was again noted at the 2-week postoperative review and remained unchanged. There was no associated skin lesion, but there was some tenderness in the area.

At the next review, 6 weeks following surgery, the pain had settled as had most of the anesthesia, leaving a small area with mild altered sensation.

## DISCUSSION

The practice of shoulder surgery is performed in either the beach-chair or the lateral decubitus position. When performing open stabilization surgery, there are advantages in alignment and access in the beach-chair position. Surgeon preference dictates that arthroscopic procedures are also often performed in the beach-chair position with axillary pouch portal.[1] In order to do this, the head must be safely supported and immobilized to control the position when it is draped out of view during surgery. There are several causes for complication in this position, with traction, joint distension and direct pressure among those most commonly noted.[2] These cases highlight neuropraxia caused by direct pressure from a hard edge of the headrest device.

The greater auricular nerve is a cutaneous branch of the cervical plexus passing almost vertically upwards along the sternocleidomastoid muscle, distributing to an area of skin on the face over the angle of the mandible, parotid gland and parotid fascia. The lesser occipital nerve is also at risk of compression by the headrest piece, with it running up the posterior border of the sternocleidomastoid to supply the posterior part of the upper neck and adjacent scalp behind the auricle. Due to its superficial anatomy, the greater auricular nerve is at risk of neuropraxia by compression, as was the cause in the cases presented here.

Arthroscopic complications were first described in 1988, with anterior staple capsulorrhaphy of the shoulder giving 5.3% complications and subacromial space surgery resulting in 0.76% complications.[2] Neurological complications due to positioning of the patient during surgery commonly involving the brachial nerve plexus related to the amount of tension on the nerves.[3] The beach-chair approach to arthroscopy has been described since early 1988 by Skyhar.[4] This patient positioning showed ease of setup and lack of brachial plexus strain.[3]

However, despite this, improper or loss of patient positioning, especially with regards to the head and neck position perioperatively, may precipitate neurological injuries.

Complications involving neuropraxia with the headrest device being the sole cause have only been reported as a case study prior to this publication, with three cases reported by Park *et al*.[5] Contributing or potentially confounding factors of patient size, occiput size and type of headrest device used in this study have been noted, along with peri- operative loss of patient positioning in one case.

**Figure 1:**
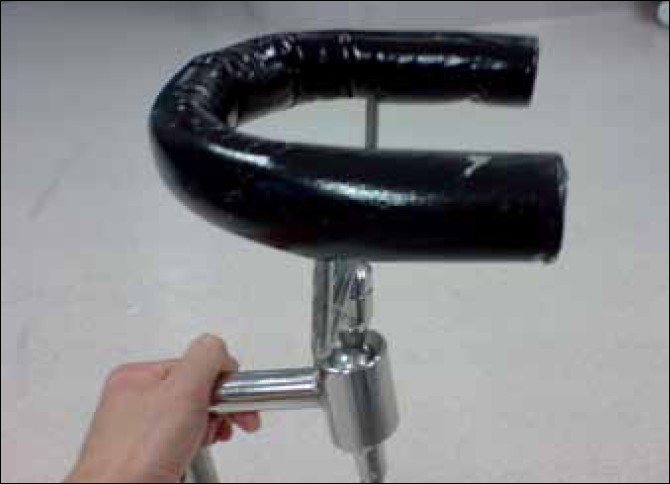
Horseshoe shaped headrest

**Figure 2:**
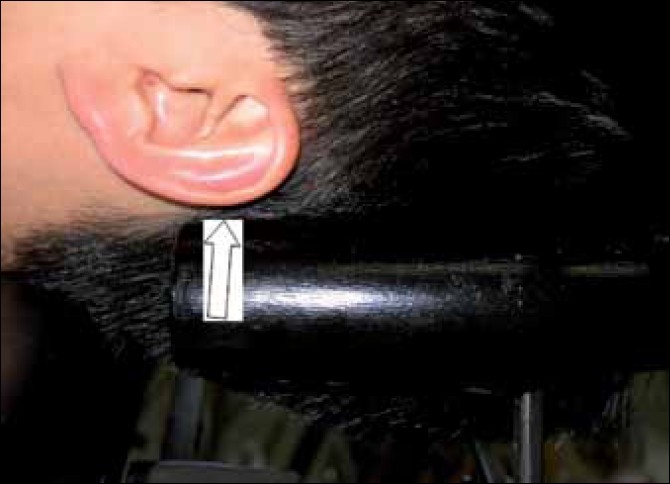
Arrow indicating compression on the greater auricular nerve

In this series, we focus more on the headrest used in the beach-chair approach as it is similar to the one described by Park *et al*.[5] The headrest is horseshoe shaped, with a black cushion covering the metal, which become prominent at the ends when the head is in position. This device is mounted on a ball and socket metallic joint that can be rotated and moved to accommodate the patient’s head in the beach chair [Figure 1]. The edge of the pillow in contact with the patient has potential to cause compression to branches of the greater auricular nerve that run superficially on top of the sternocleidomastoid muscle and postauricular region [Figure 2]. Security of positioning of the head is usually accomplished with the use of a strap attached to the headrest, which encircles the head and attaches it to the headrest. Perioperatively, the patient is given general anesthesia and is placed in the beach-chair position, which may result in loss of position of the head and neck and precipitating neurological damage.

Following these cases of resolved neuropraxia, we have ceased using this headrest device. A new headrest device consisting of a supportive pillow underneath the head has been used, with padded headstraps employed for extra stability, incorporated in a soft foam disposable mask, with no hard components that can cause direct pressure.

Careful attention to patient positioning is of paramount importance in shoulder surgery, particularly where the position is to be maintained for a prolonged period. Checking contact areas, where bony prominences may contact firm materials around the operating table and padding or eliminating these will help prevent compression and pressure injuries to the skin and soft tissues, including nerves.
